# Wearable high-density EMG sleeve for complex hand gesture classification and continuous joint angle estimation

**DOI:** 10.1038/s41598-024-64458-x

**Published:** 2024-08-09

**Authors:** Nicholas Tacca, Collin Dunlap, Sean P. Donegan, James O. Hardin, Eric Meyers, Michael J. Darrow, Samuel Colachis IV, Andrew Gillman, David A. Friedenberg

**Affiliations:** 1 Battelle Memorial Institute, Neurotechnology, Columbus, OH USA; 2 Air Force Research Laboratory, Materials And Manufacturing Directorate, Wright-Patterson AFB, OH USA

**Keywords:** Electromyography (EMG), Machine learning, Wearables, Human–robot collaboration, Biomedical engineering, Computer science

## Abstract

High-density electromyography (HD-EMG) can provide a natural interface to enhance human–computer interaction (HCI). This study aims to demonstrate the capability of a novel HD-EMG forearm sleeve equipped with up to 150 electrodes to capture high-resolution muscle activity, decode complex hand gestures, and estimate continuous hand position via joint angle predictions. Ten able-bodied participants performed 37 hand movements and grasps while EMG was recorded using the HD-EMG sleeve. Simultaneously, an 18-sensor motion capture glove calculated 23 joint angles from the hand and fingers across all movements for training regression models. For classifying across the 37 gestures, our decoding algorithm was able to differentiate between sequential movements with $97.3 \pm 0.3\%$ accuracy calculated on a 100 ms bin-by-bin basis. In a separate mixed dataset consisting of 19 movements randomly interspersed, decoding performance achieved an average bin-wise accuracy of $92.8 \pm 0.8\%$. When evaluating decoders for use in real-time scenarios, we found that decoders can reliably decode both movements and movement transitions, achieving an average accuracy of $93.3 \pm 0.9\%$ on the sequential set and $88.5 \pm 0.9\%$ on the mixed set. Furthermore, we estimated continuous joint angles from the EMG sleeve data, achieving a $R^2$ of $0.884 \pm 0.003$ in the sequential set and $0.750 \pm 0.008$ in the mixed set. Median absolute error (MAE) was kept below 10° across all joints, with a grand average MAE of $1.8 \pm 0.04^\circ$ and $3.4 \pm 0.07^\circ$ for the sequential and mixed datasets, respectively. We also assessed two algorithm modifications to address specific challenges for EMG-driven HCI applications. To minimize decoder latency, we used a method that accounts for reaction time by dynamically shifting cue labels in time. To reduce training requirements, we show that pretraining models with historical data provided an increase in decoding performance compared with models that were not pretrained when reducing the in-session training data to only one attempt of each movement. The HD-EMG sleeve, combined with sophisticated machine learning algorithms, can be a powerful tool for hand gesture recognition and joint angle estimation. This technology holds significant promise for applications in HCI, such as prosthetics, assistive technology, rehabilitation, and human–robot collaboration.

## Introduction

Human-computer interaction (HCI) is a rapidly evolving field with applications that span various domains^1,2^. Hand gesture decoding, in particular, plays a crucial role in many HCI applications, allowing for more natural and intuitive interactions with intelligent systems. In the medical domain, HCI can help facilitate assistive or rehabilitative devices that can improve the quality of life of individuals with mobility impairments. Decoded gestures could facilitate control of technologies to restore movement in individuals with motor disabilities such as prosthetics^3–8^, exoskeletons^9–16^, rehabilitation robots^17–20^, and functional electrical stimulation (FES)^21–30^. In non-medical domains, HCI is a key component in facilitating successful human-robot collaboration^31–35^. As robots become increasingly integrated into our lives, the need for effective and intuitive communication between humans and robots is imperative.

Existing human-machine interfaces (HMIs) informed by movements of the hand often use computer vision^36–38^ or kinematics measured via inertial measurement units (IMUs)^39–42^ to infer intention of the human. Computer vision approaches can be simple to setup, versatile, and cost-effective, but they rely on external sensors such as cameras or motion capture systems that can become obstructed, depend on lighting conditions, and prevent the system from being fully mobile. Kinematics-based HMIs can track physical movement, but they do not necessarily capture user intention, which can lead to a disconnect between the user’s intended action and the machine’s response^1^. As a result, many researchers have proposed using brain-computer interfaces (BCIs) to decode user intentions from neural signals to help mediate cooperation with the device or intelligent agent^19,21–29,43^. Advantages of these systems are they allow for the direct decoding of neural signals, the detection of anticipated actions^44^, and real-time updates based on implicit neural feedback^45–50^, in which both human and machine co-adapt to a shared control policy^51–53^. However, limitations with this approach include invasive neural implantation or, in the case of non-invasive BCIs, a cumbersome setup with limited information bandwidth for control^54,55^.

To address these challenges, electromyography (EMG) offers a promising and natural control signal to decode intention via neural drive to muscles. An advantage of decoding muscle activity is that neural activation of muscles can be detected approximately 50-100*ms* prior to physical movement onset^31^, allowing for low-latency devices compared to purely kinematics-based HMIs. Additionally, unlike many other interfaces, EMG does not require external sensors, such as cameras, making the interaction process more natural without requiring users to be in a particular region or orientation to use the device. Additionally, with EMG sensors locally positioned on the forearm, for example, hand movements can be decoded without occluding any hand joints. EMG can also be used as a proportional control signal, in which the magnitude of muscle activation can influence the response of the machine.

Existing EMG-based HMIs incorporate various control strategies. At the simplest level, an EMG-triggered system can deliver FES based on EMG activity crossing a predefined threshold^16^, which has shown to aid stroke rehabilitation^56^. More sophisticated methods using musculoskeletal models^9,14,15^, such as the Hill muscle model^57,58^, in combination with kinematic information, can provide continuous closed loop control based on a target muscle force estimate. However, as the HCI application complexity and degrees of freedom (DOF) increase, musculoskeletal model-based solutions can become overly complicated and impractical to implement. Instead, machine learning (ML) approaches may be more suitable to decode a large DOF using the same signals. ML based methods may also enable control of devices when the user is unable or unwilling to physically perform the action. In these cases, for instance a user who is paralyzed or is trying to covertly communicate, it may not be possible or practical to get the requisite kinematic information for musculoskeletal models via gloves or cameras. However, intention can still be discerned and characterized for HCI by decoding EMG signals using ML methods. Additional advantages of this approach are that ML can be used to both classify movements for state estimation as well as regress continuous joint positions, offering more versatile and adaptable forms of control. For instance, hand gesture classification may be useful for activating FES, triggering robotic hand grips on a prosthetic arm, or relaying state estimation for a robot to choose an appropriate action based on a learned policy in human-robot collaboration applications. Likewise, continuous joint control offers the possibility of direct end-effector manipulation, which may be a more natural HMI for the user. Furthermore, these modalities could be combined to increase the complexity and fidelity of control.

However, the effectiveness of EMG as a control signal is heavily dependent on the quality and resolution of the recorded signal. Traditional EMG systems often use a small number of manually placed electrodes, which provide limited spatial information and are highly dependent on placement. High-density EMG (HD-EMG) systems offer a solution to this by using a dense grid of electrodes to collect high-resolution signals of muscle activity, which are less susceptible to performance issues due to variation in electrode placement^59^. Typically, these systems are arranged as a compact grid patch, positioned on a single or only a few muscles. This limits their potential in applications in which the interpretation of signals from a large number of muscles is required, such as dexterous control of prosthetic limbs or in collaboration with a robot in a complex environment. With that said, recent studies have demonstrated success in using HD-EMG for control of myoelectric prosthetics and use in robotic applications^60–67^. Using a grid of electrodes can improve decoding performance and provide additional bandwidth of information to assess motor function^68^. Unfortunately, HD-EMG has historically been limited to research systems that are bulky and require significant time and expertise to setup^69^.

To address these challenges, we present the NeuroLife® EMG sleeve, a wearable garment with up to 150 embedded electrodes that spans the forearm musculature, providing a high-resolution view of muscle activity. By spanning the muscles of the forearm, the sleeve can record comprehensive muscle activity information, which can be used to decode a high number of movement classes and predict continuous joint angles, providing a versatile control signal. In this paper, we demonstrate the use of the NeuroLife EMG sleeve and discuss its potential applications in enhancing HCI. We believe that this device represents a significant step forward in the field of HCI, not only providing a more natural and intuitive form of control that can be applied in a wide range of applications, but also offering ease of use, reduced risk of electrode misplacement, and comprehensive measurements of muscles.

## Methods

### Study participants

Ten able-bodied individuals (4 female, 6 male; ages 20-35) participated in the study. EMG was recorded from their right arms as they attempted various movements and grasps. The dataset was collected as part of a study at Battelle Memorial Institute that was approved by the Battelle Memorial Institute Institutional Review Board (IRB0773). All methods implemented in this study were carried out in accordance with relevant guidelines and regulations. All participants were informed of the study protocol and provided written consent in accordance with the Declaration of Helsinki. Full participant demographics and equipment details are contained in Supplementary Table 1.

### Experimental setup and paradigm

**Figure 1:**
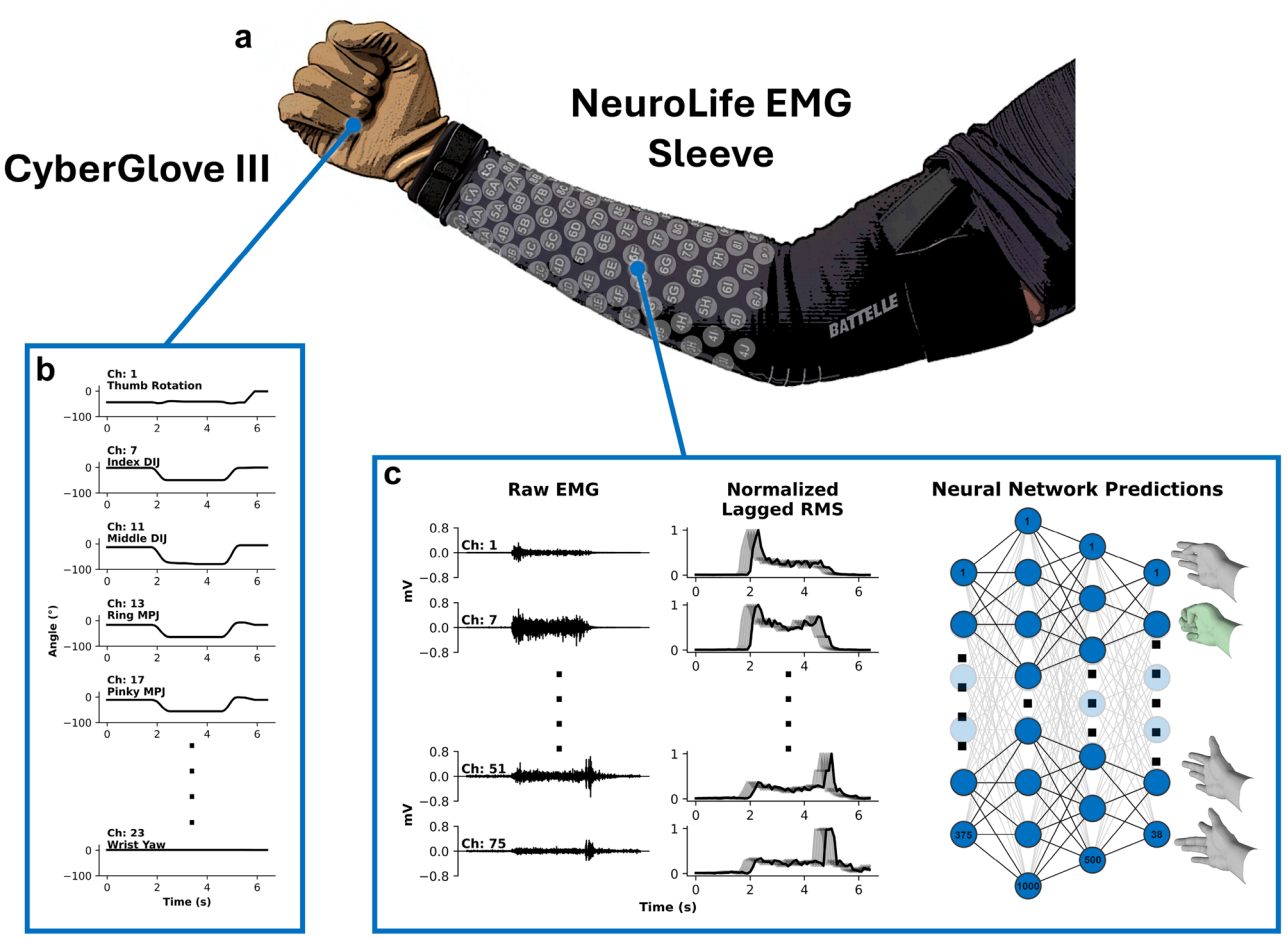
High-resolution decoding with the NeuroLife EMG sleeve. (**a**) NeuroLife EMG sleeve with up to 75 bipolar EMG channels (150 electrodes) to record muscle activity across the forearm. Participants also wore the CyberGlove III to record joint angles for regression. (**b**) Joint angles in five selected joints of the fingers during Hand Close. (**c**) Processing pipeline from filtered EMG to lagged root mean square (RMS), which is flattened as input into a linear neural network (NN). Corresponding layer sizes are seen in the bottom nodes with an exemplary correct prediction of Hand Close in green.

The experimental design is similar to previous studies^68,70^, with participants performing various hand movements and grasps as they followed cues on a monitor while EMG was recorded using the NeuroLife EMG sleeve (Fig. 1a). To enhance signal quality, the forearm was sprayed with a conduction enhancer (Signaspray, Parker Laboratories, Fairfield, NJ) prior to donning the sleeve. The sleeve comes in three different sizes, namely small (128 electrodes, 59 bipolar channels), medium (142 electrodes, 70 bipolar channels), and large (150 electrodes, 75 bipolar channels). The sleeve size was determined for each individual based on signal quality and user comfort prior to data collection (Supplementary Table 1). Sleeves were donned by aligning the zipper with the ulna. Participants also wore the CyberGlove III (Fig. 1a; Engineering Systems Technology, Kaiserslautern, Germany) which consisted of 18 bend sensors to calculate 23 joint angles (Supplementary Table 2) of the hand and fingers for regression models.

Participants were instructed to perform each movement naturally without over exertion to avoid fatigue (25-50% of their maximal effort). A foam cushion was used to prop up their arm during data collection. Data was collected using two different experimental designs we term sequential and mixed. In both sequential and mixed movement blocks, each cue was prompted for a random duration within a 2–3 second range. A subset of subjects (subjects 1, 7, 8, 9, and 10) had a preliminary baseline session (session 0), which was only used for pretraining models. All decoder testing results shown are from the main session (session 1), which was conducted for all 10 subjects.

In sequential blocks, a data block was recorded consisting of 10 repetitions of a single movement. Rest was interleaved between each of the ten attempts. A total of 37 unique movements were recorded using this structure (Supplementary Figure 1). The 10 consecutive attempts for each movement were partitioned as follows: The first four movements were used for model training, the middle three movements for model validation, and the final three movements for evaluation. Training, validation and test sets were then created by concatenating the repetitions, according to the aforementioned partitioning, for all of the 37 movements. We refer to the resulting test dataset as the “sequential” set to denote that the movements were performed in sequential order. Once model hyperparameters were fixed based on validation performance, the three validation movements were added to the training set and the models were retrained.

To account for potential bias due to the sequential cues and to simulate more complex and realistic scenarios, mixed blocks were interspersed randomly between individual movement recording blocks. Mixed blocks contained five repetitions each of 3-6 different movements. The movements were randomly shuffled within a block and rest was interleaved between movements. Mixed blocks simulate more challenging and realistic scenarios where the user is switching between several movements. Unlike sequential blocks, in the mixed blocks the participants could not anticipate the next movement cue. Some movements were repeated in multiple mixed blocks for up to 10 repetitions total of an individual movement across all mixed blocks. A total of 19 movements (Supplementary Figure 2) were included across mixed blocks for all subjects except subject 7, who only performed 17 movements. Mixed blocks were concatenated and used as an additional test set, termed the “mixed” set, to evaluate decoder performance. When training models to evaluate the mixed testing set, movements not included in mixed dataset were dropped from the training set. Additionally, the three consecutive movements that make up the sequential testing set were concatenated with the training and validation sets, for 10 attempts of each movement used for training. When using pretrained models, the mixed datasets from other subjects/sessions were used as additional training data during pretraining. In total, the lengths of the testing sets used to evaluate decoders were $9.4 \pm 0.01$ minutes for the sequential set and $11.5 \pm 0.6$ minutes for the mixed set. Refer to Supplementary Figure 3 for a summary of the experimental paradigm and data recording blocks.

### Signal processing

#### EMG system

EMG was recorded with a sampling rate of 3,000Hz using an Intan Recording Controller (Intan Technologies, Los Angeles, CA). The raw EMG signal was filtered using a notch filter at 60 Hz and bandpass filtered between 20 and 400 Hz with a 10th order Butterworth filter^68,70,71^. Filtered raw EMG from four select channels is shown in Fig. 1c (left panel). Following the filtering pipeline, root mean square (RMS) features were extracted from non-overlapping 100*ms* bins. To provide time information to the machine learning models, the 4 preceding bins were concatenated with the current bin (5 bins total). The lagged RMS was then flattened and used for model input (Fig. 1c (middle and right panels)). Lastly, RMS features were standardized using the mean and standard deviation from the training set. When pretraining, standardization was fit using the pretrained data to initialize model weights. Subsequently, when fine-tuning the model in session, standardization was re-fit using in-session training data. The full signal processing pipeline was implemented using sklearn pipelines^72^ to avoid any data leakage during processing.

#### Joint angles

**Figure 2:**
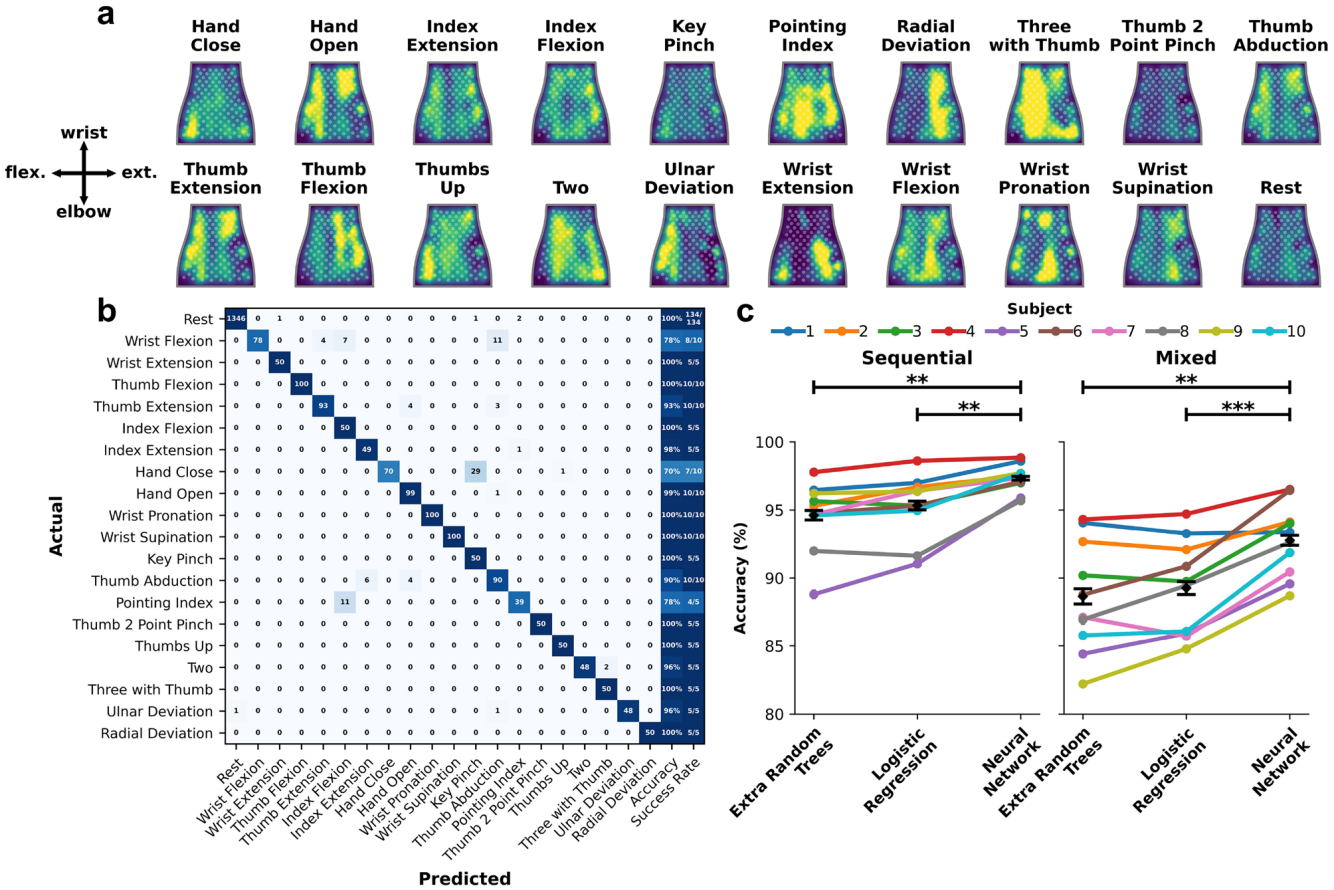
Classifying hand movements and grasps using the NeuroLife EMG sleeve. (**a**) Average normalized RMS heatmaps of EMG activity during movements attempted in the mixed dataset mapped to the flattened NeuroLife EMG sleeve. (**b**) Exemplary confusion matrix from mid-window decoding of the mixed dataset for subject 1. Total bin-wise accuracy and success rate by movement are shown in the last two columns (**c**) Full decoding performance from the mid-window analysis across all subjects and models. The NN outperforms both the extra random decision trees and logistic regression models.

Joint angles were calculated from strain sensors embedded in the CyberGlove III that recorded data with a sampling rate of 90Hz and were resampled to 10Hz time-synced with EMG RMS bins (1 joint angle vector sample per RMS bin). The CyberGlove III used consisted of 18 strain sensors that measured the metacarpophalangeal joint (MPJ), the proximal interphalangeal joint (PIJ), and the abduction joint for all fingers, in addition to thumb rotation, thumb abduction, palm arch, wrist pitch, and wrist yaw joint angles directly (Supplementary Table 2 and Figure 2). The distal interphalangeal joint (DIJ) for each finger and thumb interphalangeal joint (IJ) were calculated based on 30% of the MPJ joint angle squared of the same finger/thumb. To smooth joint angle predictions, a moving average of the 4 preceding bins and the current bin was used for the ground truth in regression modeling. Smoothed angles from six select joints are shown in Fig. 1b. After an initial baseline session with subject 1, an index strain sensor broke, affecting the index MPJ and index DIJ angles. Therefore, in all group regression analyses, these two joint angles were removed for a 21 target regression task to avoid biasing results. Figure 6d and Supplementary Video 2 show exemplary results of the full 23 joint regression task from subject 1’s baseline session prior to the sensor defect. For visualizations, the CyberGlove III joint angles were mapped to a Unity-based virtual hand (Supplementary Table 2). It should be noted that the virtual hand does not support ab/ad-duction of the fingers. Therefore, in movements such as “Fingers Together”, the virtual hand visualization does not show the fingers touching, but the CyberGlove III records finger ab/ad-duction.

### Hand pose classification

Models were trained to classify movements using the training data and evaluated on both the sequential and mixed testing datasets. To assess how much training data was required for adequate decoding performance, movements were sequentially added from the training dataset. Except where explicitly noted, group decoding results use the maximum amount of training data available. For each training condition, five models were trained using five different random seeds. Each model used the same training data and was evaluated on both sequential and mixed datasets. Performance metrics were averaged across the five repetitions to characterize performance and variability.

The classification models evaluated consisted of an extra random decision trees^73^, logistic regression^74–78^, and a neural network (NN)^79^. Input to the classification models was consistent across all models used (N channels x 5 bins = 375 input features for the large sleeve). For the extra random decision trees and logistic regression classifiers, the default parameters from scikit-learn were used. The NN architecture used was the same as in previous studies^70^ with two fully connected hidden layers of 1,000 and 500 units. This NN structure was chosen due to its robustness in time-series classification and ability to achieve high performance in subject-specific decoding use-cases. Batch normalization and the ReLU activation function were used between layers. The final layer had a size of 38 (sequential) or 20 (mixed) to account for all movements plus rest. A softmax function was applied to the last layer to obtain prediction probabilities of each class. To prevent overfitting, dropout was applied to each layer with 20% probability. The learning rate was optimized using the FastAI learning rate finder tool^80^. Label smoothing cross entropy loss (p=0.9) and the Adam optimizer^81^ were used for training. Each model was trained for 200 epochs using the one cycle training policy from FastAI^82^. With the exception of the pretrained models, neural network models were randomly initialized using default settings^80^.

When pretraining models, RMS features across different sleeve sizes of participants were mapped to the medium sleeve for a total of 70 channels across subjects^68^. All other subject data aside from the test subject were used to pretrain models. Once pretrained, models were fine-tuned within session using 50 epochs.

Two scenarios were used to evaluate decoders, namely a mid-windows analysis and a simulated real-time (continuous) analysis. In the mid-windows analysis, the middle 1-second window of cues were used in both training and testing datasets^70,83,84^. This analysis demonstrates whether or not decoders are able to differentiate the movements, but ignores transition periods. To assess the potential for real-time decoding ability, inference time of the full filtering to model prediction pipeline was evaluated on data collected with the large sleeve (75 channels). System inference was calculated on each successive 100*ms* bin of raw EMG over 10 repetitions of subject 1’s sequential test set. A HP Z-book with 2.50*GHz* 11${\text {th}}$ Gen Intel Core i7 processor was used for calculating inference. Additionally, a continuous analysis was conducted in which the full time-series data was used to assess a decoder’s ability to classify movements and movement transitions. Various static and dynamic temporal cue shifts were used to account for reaction time of subjects responding to visual cues. The dynamic cue shifting technique used was based on previous work^70^ that uses a minimum residuals sweep algorithm to locate the steepest slope of RMS activity near the onset and offset of a cue to align cues with muscle activity changes.

**Figure 3:**
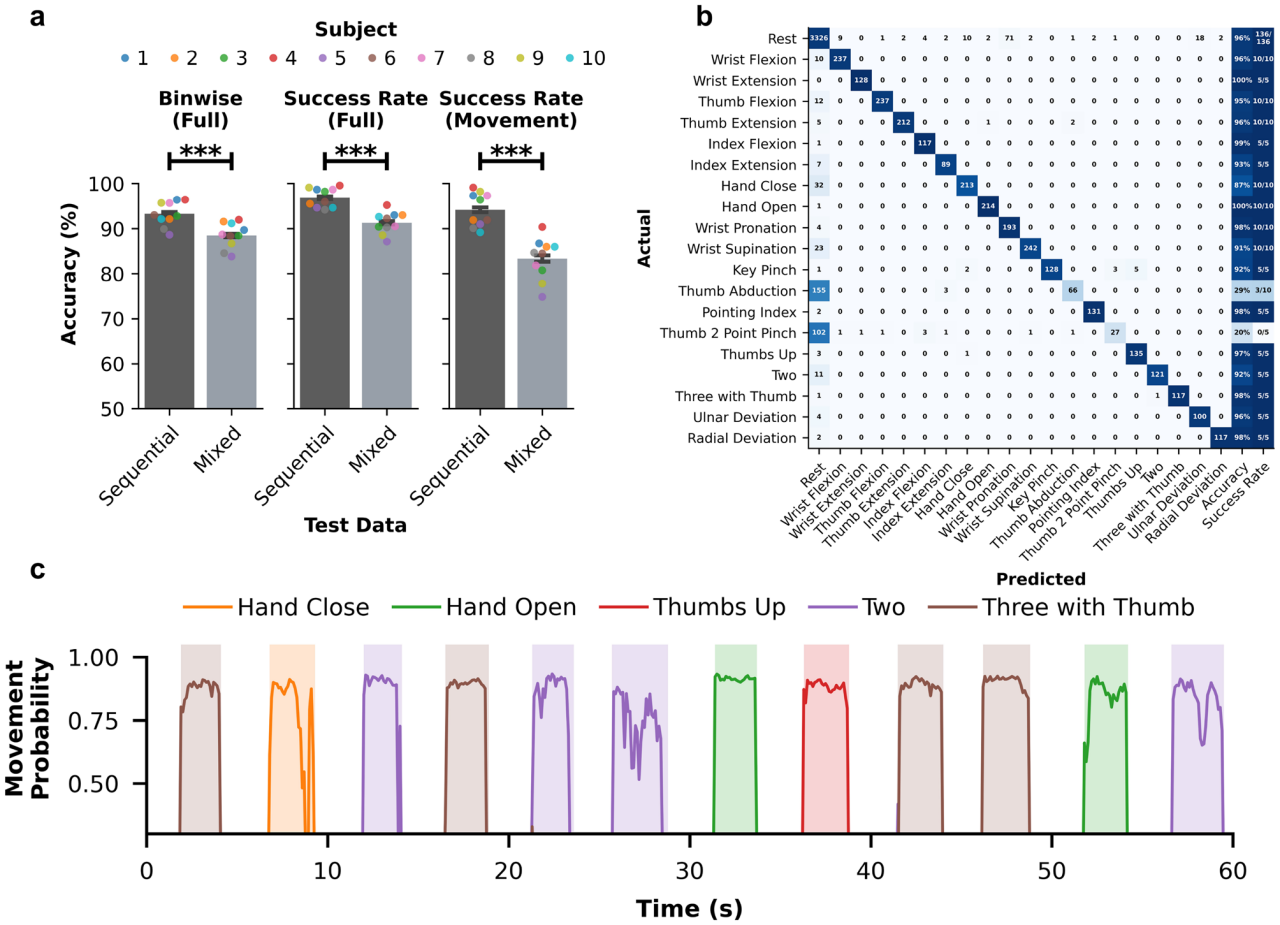
Continuous classification using the NeuroLife EMG sleeve. (**a**) Full decoding results using the dynamic cue shifting method across all subjects and datasets. Bin-wise accuracy, success rate, and movements-only success rate demonstrate high decoding performance in a simulated real-time scenario. (**b**) Exemplary confusion matrix from continuous decoding of the mixed dataset for subject 4. Total bin-wise accuracy and success rate by movement are shown in the last two columns (**c**) Exemplary simulated real-time prediction probability plot from subject 4 across a 1 minute snippet of data from the mixed dataset. The predictions match the cues with a slight onset delay for some movements.

To evaluate decoding performance, bin-wise accuracy and success rate metrics were used. Bin-wise accuracy consisted of the percentage of 100*ms* bins that match the ground truth. Since the rest class was interleaved between all movements, chance accuracy was 50% (i.e., a naive decoder that only predicted rest would obtain approximately 50% accuracy). Success rate, on the other hand, was used to evaluate decoders at the movement level, scoring whether or not the decoder responded appropriately to the cued movement. The success rate metric is meant to approximate an observer scoring each cued movement as a binary success or failure. To count as a successful movement, the decoder needed to satisfy two criteria: 1. predict the correct movement over 5 consecutive bins (0.5 seconds) within the cue duration, and 2. predict the correct movement for at least 50% of the cue duration. Similar to the bin-wise accuracy, chance level for success rate was 50% because each movement cue had a corresponding rest cue. When calculating success rate by movement in which rest was excluded and only movements were considered (Fig. 3a; right), chance level was 2.7% and 7.6% for the sequential and mixed datasets, respectively.

**Figure 4:**
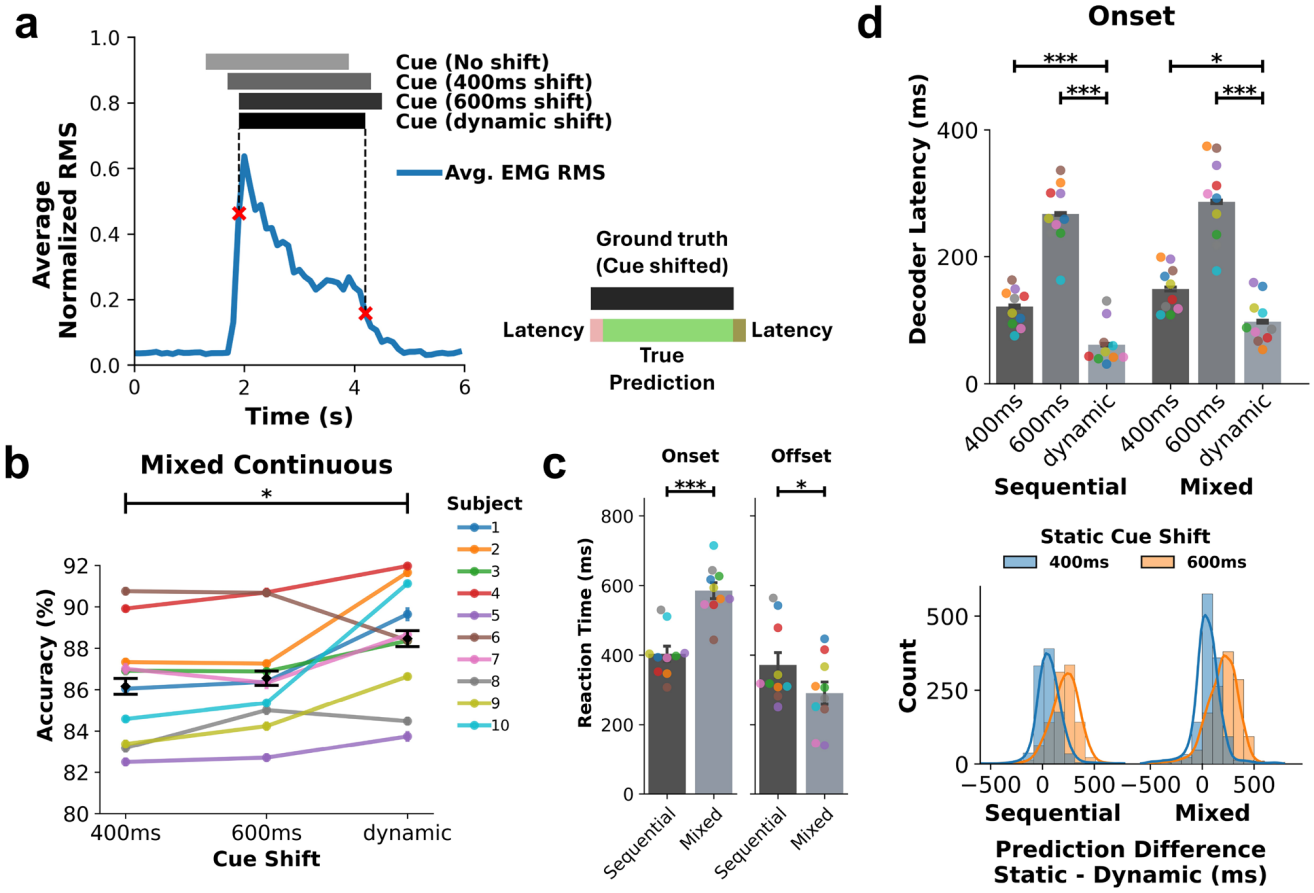
Comparing cue shift techniques during continuous decoding. (**a**) Left: Schematic of static cue shifts and reaction time calculation based on RMS onset/offset for dynamic cue shifting. Cues are either shifted by a static amount or dynamically based on when RMS activity slope is steepest using a minimum residuals sweep algorithm. Right: Decoder latency of predictions (green bar) with respect to the ground truth labels/shifted cues (black bar). (**b**) The dynamic cue shifting technique accounts for varying reaction times across subjects yielding higher decoding performance than the static 400*ms* cue shift results in the mixed dataset (**c**) Average reaction time calculated via the dynamic cue shifting algorithm. Each point represents the average reaction time per subject. Subjects took longer to react to movement cues in the mixed dataset compared to the sequential dataset made up of consecutively repeating movements. (**d**) Top: Decoder latency with respect to muscle activity onset across the different cue shifting methods demonstrates reduced latency when using the dynamic cue shifting technique. Bottom: Distribution of the difference between decoder prediction onsets trained with static and dynamic cue shifts per movement attempt.

Decoders were also evaluated based on how fast they responded to user intentions. A decoder latency was calculated in which the first time sample of the correct prediction with respect to a common reference was used to assess prediction lag. In Fig. 4d, reaction time based on EMG RMS activity was used as the reference point to determine latency with respect to the user’s muscle activity onset. The difference between predictions made from decoders trained with static and dynamic cue shifts were compared across all movement attempts. Supplementary Figure 4 provides a summary of the classification analyses performed.

### Hand joint angle regression

To determine whether hand poses via joint angles could be continuously predicted using the sleeve, regression models were trained on the same training data over an increasing number of attempted movements and evaluated on both sequential and mixed datasets. Three regression models were used, namely ridge^85^, laplacian kernel ridge^86^, and a linear NN. Default parameters from scikit-learn were used for both the ridge and laplacian kernel ridge models. The NN architecture used was similar to the classification NN except for the addition of one additional hidden layer directly after the input layer with a size of 4,000. Dropout probability in each of the 3 hidden layers was increased to 40% to avoid overfitting. The final layer was mapped to the number of joint angles with values truncated between the minimum and maximum joint angles determined from the training set. Similar to the classification model, the NN was trained with a one cycle policy with an optimized learning rate using the learning rate finder tool and Adam optimizer. The mean squared error between predicted and ground truth joint angles was used as the loss function during training. The coefficient of determination ($R^2$) between predicted and ground truth joint angles was used to evaluate regression model performance since it only generates a high score if the majority of the ground truth group has been predicted correctly^87^. Additionally, to understand the relative magnitude of error by joint, we computed the median absolute error (MAE) across all joint angles. Refer to Supplementary Figure 4 for a summary of the regression analyses performed.

### Statistical analysis

Data distributions were tested for normality using Lilliefors tests. Multiple statistical comparisons were made on normally distributed data using a oneway ANOVA to determine whether the true means underlying each sample were identical. Post-hoc pairwise (Figs. 2c & 4b) and single pairwise (Figs. 3a & 4c) statistical comparisons were determined using paired t-tests (Figs. 2c & 4b–c), and were planned a priori. For the non-normally distributed decoder latency results, a non-parametric Kruskal-Wallis H-test was used to compare the population median between groups. Post-hoc pairwise statistical comparisons were made using Dunn’s test (Fig. 4d). All statistical tests were calculated using samples that consisted of the average across the 5 seeds per training condition for all analyses. An alpha of 0.05 was used to determine significant differences for single comparisons. To correct for multiple post-hoc pairwise comparisons, a Bonferroni-corrected alpha was used in which the alpha was divided by the number of comparisons ($n_{comp}$) made. In Fig. 2c, the NN was compared with the extra random decision trees and logistic regression models ($n_{comp}=2$). In Figs. 4b and d the dynamic cue shifting method was compared with both static 400 and 600*ms* cue shifts ($n_{comp}=2$). Statistical tests for each comparison are noted in the text. Statistical analysis was performed in Python 3.8 using SciPy^88^ and scikit-posthocs^89^. In all figures, * indicates *p* < 0.05/$n_{comp}$, ** indicates *p* < 0.01/$n_{comp}$, and *** indicates *p* < 0.001/$n_{comp}$. Error bars indicate mean ± standard error of the mean (SEM) in all figures.

## Results

### High-resolution EMG for hand pose classification

We first assessed whether the EMG sleeve can accurately predict a range of movements and grasps irrespective of transition periods. Similar to methodology used in other EMG studies^70,83,84^, the middle 1 second window of each cue was extracted and used for either model training or evaluation as per the data splits described in the methods. Figure 2a shows average normalized RMS heatmaps by movement for subject 1 from the mixed dataset, showing the sleeve’s ability to obtain a high-fidelity EMG signal across the muscles that span the forearm. The spatial EMG heatmaps highlight active muscle areas that correspond with the intended movement as well as show average differences between movements. EMG classification models were able to distinguish between the movements with high accuracy (Fig. 2c). An exemplary confusion matrix of the NN classification results from subject 1 from the mixed dataset is shown in Fig. 2b. The decoder is able to correctly predict the 20 classes with very few errors. In this example, model confusion arises from Hand Close and Key Pinch, which are two very similar movements with slight differences in thumb positioning (Supplementary Figure 1). Additional confusion arises from Wrist Flexion and Thumb Abduction, as well as one error confusing Pointing Index with Index Flexion, resulting in 6 misclassified movements total out of 135 for a success rate of 95.6%.

Classification model performance was statistically different across the three models investigated over both testing datasets ($F[2,7] = 5.60; p = 0.009$). Averaging across all participants, the NN achieved a bin-wise accuracy of $97.3 \pm 0.3\%$ in the sequential dataset, outperforming both the LR ($95.3 \pm 0.7\%; p=1.9\times 10^{-3}$) and extra random decision trees ($94.6 \pm 0.8\%; p=8.0\times 10^{-4}$) models (Fig. 2c; left). The NN also outperformed both models in the mixed dataset, achieving a bin-wise accuracy of $92.8 \pm 0.8\%$, with the LR and extra random decision trees achieving $89.3 \pm 1.1\% \;(p=1.6\times 10^{-4})$ and $88.6 \pm 1.3\% \; (p=7.1\times 10^{-4})$, respectively (Fig. 2c; right). Average success rate of the NN model’s ability to identify movements and rest periods on a full cue level was $98.3 \pm 1.0\times 10^{-3}\%$ for the sequential set and $93.7 \pm 3.6\times 10^{-3}\%$ for the mixed set. Ignoring rest periods, the NN achieved $96.6 \pm 1.8\times 10^{-3}\%$ and $87.6 \pm 7.1\times 10^{-3}\%$ success rate for the sequential and mixed datasets, respectively, showing the ability to reliably differentiate muscle activity of different movements. Refer to Table 1 for a summary of NN decoding results across all conditions.

**Table 1:** Full decoding results across datasets and analyses.

Test dataset (analysis)	Classification			Regression	
	Bin-wise accuracy (%)	Success rate (%)	Movements-only success rate (%)	Coefficient of determination ($R^2$)	Median absolute error (MAE)
Sequential (mid-windows)	$97.3 \pm 0.3$	$98.3 \pm 1.0\times 10^{-3}$	$96.6 \pm 1.8\times 10^{-3}$	N/A	N/A
Mixed (mid-windows)	$92.8 \pm 0.8$	$93.7 \pm 3.6\times 10^{-3}$	$87.6 \pm 7.1\times 10^{-3}$	N/A	N/A
Sequential (continuous)	$93.3 \pm 0.9$	$96.9 \pm 0.7$	$94.2 \pm 1.2$	$0.884 \pm 0.003$	$1.8 \pm 0.04^\circ$
Mixed (continuous)	$88.5 \pm 0.9$	$91.3 \pm 0.8$	$83.3 \pm 1.5$	$0.750 \pm 0.008$	$3.4 \pm 0.07^\circ$

### Continuous classification to simulate real-time use

While being able to classify movements on a gross level highlights the sleeve’s ability to differentiate muscle activity from a variety of hand poses, for a system to be usable in real-time applications, decoders should have fast inference speed and be able to classify movements continuously, including transitions between states. Inference time of each 100*ms* bin of raw EMG data passed through the full filtering to NN model prediction pipeline with the large sleeve (75 channels) was $2.7 \pm 0.015ms$ per sample, well under the 100*ms* bin length, suggesting the potential to classify movements in real-time. To determine whether decoders could adequately classify movements and movement transitions, models were trained on the full continuous signal and evaluated on the full sequential and mixed testing sets (Fig. 3). Using the dynamic cue shifting technique to account for participant reaction time to cues, the NN model achieved a bin-wise accuracy of $93.3 \pm 0.9\%$ on the sequential set and $88.5 \pm 0.9\%$ on the mixed set ($p=2.3 \times 10^{-4}$). When evaluating success rate on the full cue level, the NN model achieved $96.9 \pm 0.7\%$ and $91.3 \pm 0.8\%$ on the sequential and mixed datasets, respectively ($p=1.3 \times 10^{-4}$). Considering only movements and ignoring rest periods, the NN model achieved a success rate of $94.2 \pm 1.2\%$ for the sequential set (chance: 2.7%) and $83.3 \pm 1.5\%$ for the mixed set (chance: 7.6%), demonstrating the ability to differentiate both movements and movement transitions via EMG activity ($p=1.8 \times 10^{-4}$).

An exemplary confusion matrix for subject 4 from the continuous mixed dataset is shown in Fig. 3b. There is minimal to no confusion between two movement classes with most of the errors occurring between a movement and rest. In this particular case, the decoder predicted rest instead of Thumb Abduction and Thumb 2 Point Pinch. Across the other movements cued, the decoder predicted rest for some time bins near the transitions between movement and rest cues, suggesting a potential decoder latency or lag due to reaction time around cue transitions.

To demonstrate what real-time classification would look like, a simulated continuous prediction probability of decoder output is shown in Fig. 3c. Movement probabilities determined by the softmax output of the NN are shown over time, predicted every 100*ms*. In this 1 minute segment of the mixed dataset from subject 4, the decoder predicted each movement correctly based on the dynamically shifted cue ground truth (shaded regions) with minimal to no lag in response time. Supplementary Video 1 shows the full simulated real-time decoding output.

### Decoder latency can be minimized by accounting for reaction time

When using EMG devices in real-time applications, decoder latency should be minimized for natural control. Here, we assessed whether decoder latency can be minimized, and subsequently improve decoding performance and responsiveness of the system by accounting for reaction time. Figure 4a shows a schematic of normalized RMS activity in response to a Hand Open cue. The original cue (light gray bar) given to participants is shifted to the right in time (darker gray bars) to account for the participant’s response time to the visual cue and time to volitionally perform the movement. A static shift in time to the ground truth labels for supervised model training can help compensate for an average reaction time to cue onset/offset. Another method to account for reaction time is to dynamically shift cues based on EMG activity. In this case, reaction time is determined by extracting time points of the steepest slope of RMS onset and offset (red $\times$), thereby dynamically shifting the cues to align with muscle activity. A schematic showing predictions (green bar) with respect to ground truth labels (black bar) shows how the decoder latency metric is calculated.

Shifting cues did not have a significant effect on decoding performance ($F[2,7] = 2.03; p = 0.15$). However, within subject, decoding performance improved in the mixed dataset in all subjects except subject 6 (Fig. 4b) when using a dynamic cue shifting method to align cue transitions with EMG activity, indicative of intentional movement, over a static cue shift of 400*ms* ($86.2 \pm 0.4\%; p=1.3\times 10^{-2}$). When comparing the dynamic cue shifting method with a static 600*ms* method, subjects 6 and 8 had higher decoding performance with the static cue shift, and there was no overall significant difference in decoding performance ($86.6 \pm 0.3\%; p=2.8\times 10^{-2}$). When comparing sequential and mixed sets, subjects reacted slower to the onset of cues ($p=1.4\times 10^{-7}$) in the mixed set ($585 \pm 23ms$) compared to the sequential set ($404 \pm 22ms$). However, in the mixed set, participants returned to rest faster ($p=2.2\times 10^{-2}$) during mixed blocks ($290 \pm 32ms$) compared to individual movement blocks making up the sequential set ($371 \pm 36ms$).

Decoder latency with respect to reaction time was affected by the cue shifting method (Fig. 4d; $H[2,7] = 44.3; p=2.39 \times 10^{-10}$). As the cue shift increased, prediction latency for the onset of movements increased, since the ground truth label over-shifted with respect to the onset of EMG activity. The dynamic cue shift method reduced decoder latency ($61 \pm 10ms$) compared to both static 600*ms* ($266 \pm 16ms;$ $p=1.6\times 10^{-4}$ ) and 400*ms* ($120 \pm 9ms;$ $p=1.9\times 10^{-3}$ ) shifts at the onset of movements in the sequential set. In the mixed set, decoder latency at the onset of movements was significantly lower when using the dynamic cue shift method ($99 \pm 11ms$) compared to a static 600*ms* shift ($289 \pm 21ms;$ $p=1.6\times 10^{-4}$ ) and 400*ms* shift ($149 \pm 11ms;$ $p=0.010$ ). Figure 4d (bottom) shows the distribution of the difference between predictions made with static and dynamic shifts, respectively across all movement attempts by subjects. Decoders trained using the dynamic cue shifting method responded faster, with 92.5% and 83.0% of predictions occurring earlier or at the same time than predictions made from decoders trained with static 600*ms* and 400*ms* cue shifts, respectively.

### Pretrained models can help reduce the amount of in-session calibration data

**Figure 5:**
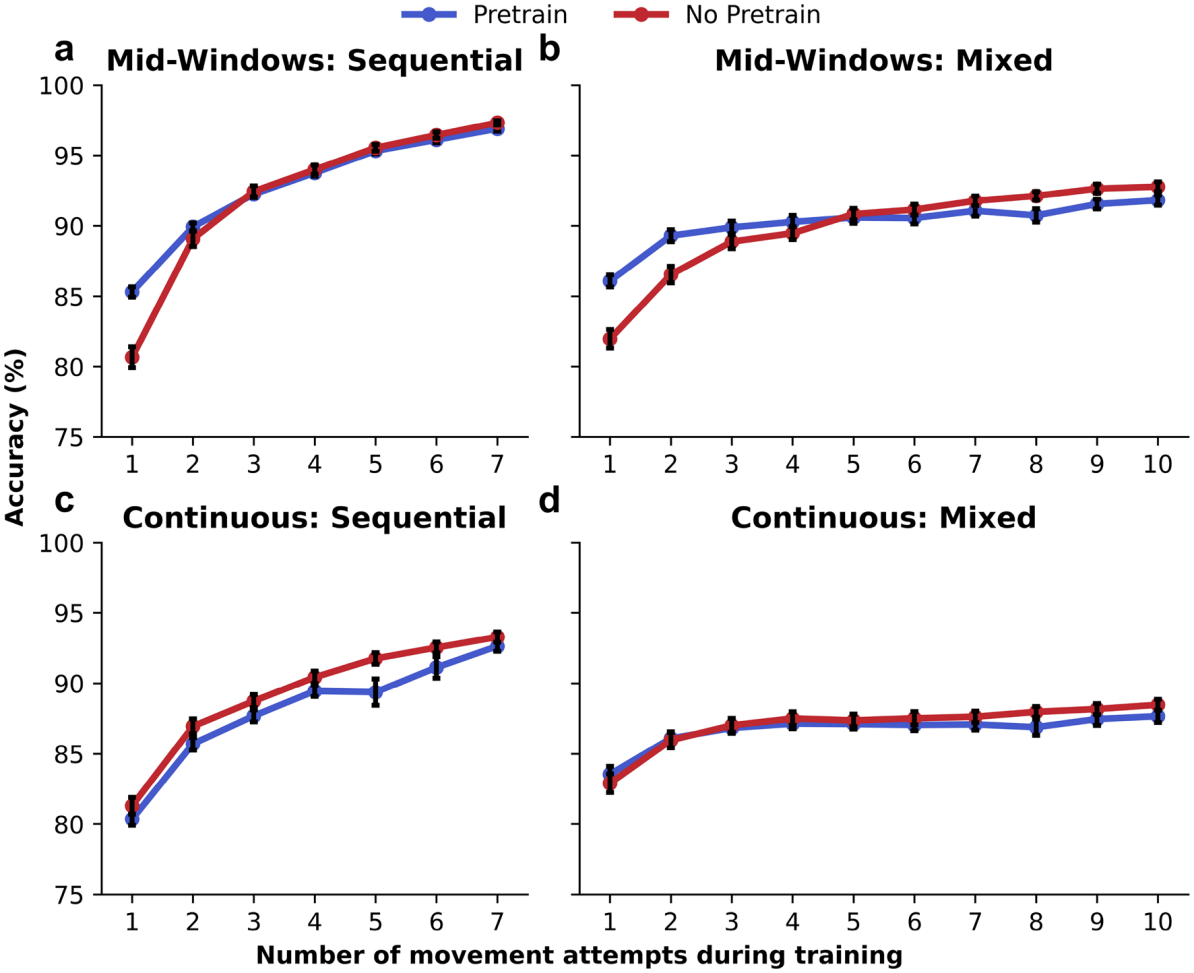
Pretraining models can increase performance when reducing the amount of training data. (**a**–**d**) Comparing pretrained (blue) and non-pretrained (red) models across an increasing number of attempted movements during training for both mid-windows (**a**) and (**b**) and continuous (**c**) and (**d**) analyses in both sequential and mixed datasets. When reducing the number of movements in training to just one attempt of each movement, the pretrained models outperformed non-pretrained models in the mid-window analysis. As the number of movements within a session increases, it is advantageous to train a new model using the in-session data only with the proposed decoding pipeline.

Reducing in-session training time can help make EMG devices easier and faster to use^90,91^. One potential method for reducing the amount of in-session training data needed is to leverage historical data to pretrain a NN’s model weights. Subsequently, in-session data is used to fine-tune the pretrained weights. To evaluate whether pretraining models from other subjects and other sessions (if available) could help reduce training data requirements while retaining high decoding performance, we compared decoders that were pretrained to decoders that were not over an increasing number of attempted movements in the training set (Fig. 5). By using the same NN architecture and without taking into account subject or session information, the simple pretrained decoders outperformed the decoders trained on in-session data when there were only a few movements in the training set. As the number of movements in the session increased, the non-pretrained decoders tended to outperform the pretrained decoders. These trends are evident in the mid-window assessment for both the sequential and mixed datasets (Fig. 5a and b). When reducing down to just one attempt of each movement, pretrained decoders increased decoding performance from $80.7 \pm 0.7\%$ to $85.3 \pm 0.4\% \; (p=2.5\times 10^{-11})$ in the sequential set and from $82.0 \pm 0.7\%$ to $86.1 \pm 0.4\% \; (p=9.5\times 10^{-11})$ in the mixed set. In the continuous case, however, the pretrained models did not help improve decoding performance. While it appears there may have been a slight boost in performance for the mixed block when reducing down to only one movement attempt, there was no significant difference between the two approaches. This was also evident in the sequential set, which favored non-pretrained decoders to adequately handle movement transition periods.

### Predicting continuous joint angles

**Figure 6:**
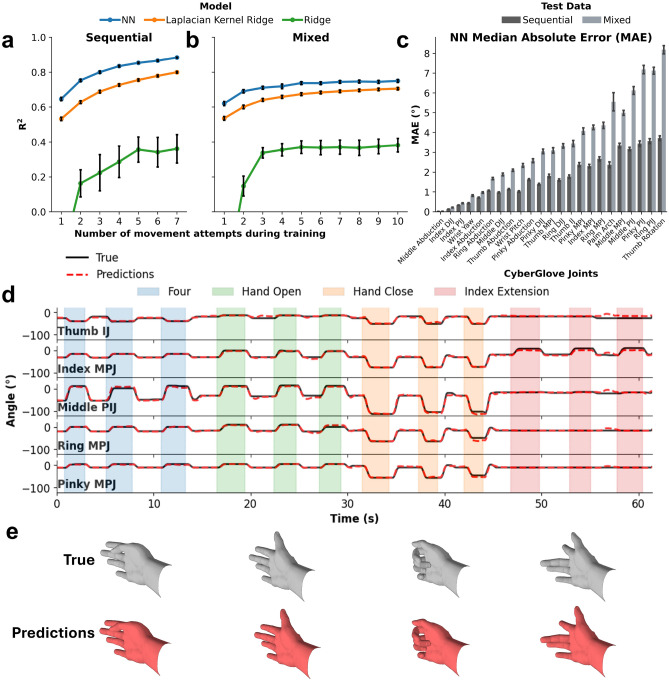
Regressing joint angles using the NeuroLife EMG sleeve. (**a** and **b**) Regression results across an increasing number of attempted movements during training comparing different regression models. The NN was able to achieve a greater $R^2$ than laplacian kernel ridge and ridge regression models in both the sequential and mixed testing datasets. **c.** Median absolute error (MAE) of joint angles predicted by the NN model in both sequential and mixed datasets. The NN was able to predict joint angles within 10$^\circ$ of the ground truth across all joints. **d.** Exemplary simulated real-time joint angle predictions (red-dashed) compared to the ground truth (black) joint angles in a one minute snippet of data from the sequential dataset of subject 1. (**e**) Snapshots of a virtual hand comparing the predicted joint angles with the ground truth joint angles across time for the four cued movements shown.

To evaluate whether the NeuroLife EMG sleeve could continuously predict hand and finger position across the various movements in both sequential and mixed datasets, we trained regression models to predict joint angles calculated by the CyberGlove III (Fig. 6). Similar to the classification paradigm, we trained regression models with an increasing number of attempted movements from the training set to determine how much calibration data is required to achieve high-performance joint estimations (Fig. 6a and b). Across all training conditions varying the number of attempted movements, the NN outperformed both the laplacian kernel ridge and ridge regression models in both the sequential and mixed datasets. When considering the full training dataset, the NN was able to achieve a $R^2$ of $0.884 \pm 0.003$ in the sequential set and $0.750 \pm 0.008$ in the mixed set. As the number of movement attempts in the training set increased, $R^2$ also increased, plateauing around 5 movement attempts for the mixed set (Fig. 6b). When training on the full training set, the NN had minimal joint angle deviations when compared to the ground truth. Errors across joints and datasets varied with the Thumb Rotation joint having the largest MAE. Average MAE was held below $10^\circ$ for all joints in both sequential (Avg. MAE: $1.8 \pm 0.04^\circ$) and mixed (Avg. MAE: $3.4 \pm 0.07^\circ$) datasets.

Figure 6d shows an exemplary simulated real-time plot of select joint angle predictions compared with ground truth measurements in a one minute snippet from the mixed dataset from subject 1’s baseline session (session 0). The full simulated real-time joint angle predictions across all joints for this test set are shown in Supplementary Video 2. Corresponding virtual hand poses mapped from the ground truth and predicted joint angles are shown in Fig. 6d. The virtual hand poses match closely with the true poses across the full dataset, demonstrating the feasibility of using the NeuroLife EMG sleeve for real-time position estimation.

## Discussion

Results from this study demonstrate the potential of the NeuroLife EMG sleeve to improve HCI by providing a wearable high bandwidth interface for the user. The sleeve is able to record high-fidelity EMG from muscles spanning the forearm, enabling high decoding performance across a wide range of movements and grasps. We demonstrate the ability for the sleeve to discern between different movements and movement transitions for use in real-time applications. Additionally, a key contribution of this work is determining how supervised training conditions affect decoder latency. By considering cue shifts and user reaction time, we were able to minimize decoder latency, thereby improving the responsiveness of the system. The study also presents a proof of concept for using pretrained models to reduce calibration time. With further refinements, this approach could make EMG devices more user-friendly and accessible by reducing the setup time. Finally, the study evaluated the capability of the system to continuously estimate joint angles using regression. This capability could be particularly beneficial in the development of advanced prosthetics, virtual reality applications, rehabilitation, and interaction with intelligent agents.

In this study, both a sequential set made up of repeated individual movement attempts and mixed set made up of randomly presented movements were used to evaluate decoders. Despite decoding fewer classes in the mixed set, decoder performance was consistently higher in the sequential test set. Potential reasons for this discrepancy include the temporal proximity to the training data, differences in training paradigm and testing paradigm, additional number of attempts of each movement in the mixed set, and the subject’s anticipation (or lack thereof) of upcoming cues. These differences in the datasets highlight a limitation in the experiment design and suggest that the system’s performance may vary depending on the how well the training paradigm reflects the inference scenario. Despite slightly lower performance compared to the sequential set, we demonstrated that decoders were able to discriminate movements in the mixed dataset with reliable decoding performance.

Our work expands upon other studies in the field of EMG pattern recognition. Refer to Table 2 for comparative works. In the classification task, many existing studies focus on a limited number of classes^1,2^, and typically do not report a full bin-wise accuracy for a sense of performance in real-time scenarios. Rather, accuracy of gross movement attempts is presented. In contrast, our study demonstrated high bin-wise decoding accuracy across a wide range of movements, demonstrating its potential use in real-time applications. While our experimental paradigm includes rest periods between movements, yielding 50% chance accuracy, we also report a success rate metric by movement only (ignoring resting cues) in which the NN achieved $96.6 \pm 0.0\%$ in the sequential set and $87.6 \pm 0.0\%$ in the mixed set, providing a more comparable metric to other works. In terms of regression, our study achieved similar performance to other works (Table 2), while predicting across a wider range of movements and joints. This suggests that our approach could potentially offer more nuanced control of robotic hands, exoskeletons, or complex FES patterns.

**Table 2:** Comparative studies assessing surface EMG decoding of hand and wrist gestures (NN: neural network, SVM: support vector machine, LDA: linear discriminant analysis, NMF: non-negative matrix factorization, LSTM: long-short term memory, CNN: convolutional neural network, MAFN: multi-attention feature fusion network, CER: classification error rate).

Reference	Number of subjects	Number of EMG channels	Number of gestures	Number of joints	Model	Results
*This work*	10	$59-75$	37/19	23	$\textrm{NN}$	Accuracy: $88.5-97.3 \%$ $R^{2}: 0.75-0.88$
Matsubara and Morimoto 2013^92^	11	4	5	N/A	SVM	Accuracy: $73 \%$
Al-Timemy et al. 2013^93^	10	6	15	N/A	Orthogonal fuzzy neighborhood discriminant analysis / LDA	Accuracy: $98 \%$
Li et al. 2013^94^	6	6	7 isometric	N/A	Boosted random forests	Accuracy: $92 \%$
Pan et al. 2014^95^	6	7	7	2	LDA	CER: $6.2 \%$ $R^{2}: 0.84$
Riillo et al. 2014^96^	20	6	5	N/A	NN	Accuracy: $89.4 \%$
Naik and Nguyen 2014^97^	8	2	5	N/A	NMF / NN	Accuracy: $87.6-93.4 \%$
Li et al. 2014^98^	10	4	4	N/A	$\textrm{NN}$	Accuracy: $93 \%$
Liu et al. 2015^99^	5	4	11	N/A	Invariant feature extraction / linear discriminant analysis	Accuracy: $91.2 \%$
Stango et al. 2015^62^	7	48	9	N/A	Support vector machine	Accuracy: $95.8 \%$
Jiralerspong et al. 2017^100^	12	6	17	N/A	NN	Accuracy: $83 \%$
Sezgin 2019^101^	42	2	5	N/A	Extreme learning machine	Accuracy: $92.8-98.9 \%$
Chen et al. 2020^102^	11	192	11	N/A	Motor unit decomposition / SVM	Accuracy: ${>}95\%$>95%
Anam et al. 2020^103^	10	16	41 (NinaPro^104^ DB5)	8	LSTM	$R^{2}: 0.87$
Lee et al. 2021^105^	10	3	10	N/A	$\textrm{NN}$	Accuracy: $94 \%$
Sri-iesaranusorn et al. 2021^106^	10/22	8/16	41 (NinaPro DB5 / DB7)	N/A	NN	Accuracy: $84.0-93.9 \%$
Chen et al. 2021^107^	10/1	8	41 (NinaPro DB5) / 30	N/A	MultConvEMG	Accuracy: $92.8 \%/\,95\%$
Lin et al. 2022^108^	1	16	12	N/A	Evidential CNN	Accuracy: $76.3 \%$
Guo et al. 2023^109^	38	12	28 (NinaPro DB2)	22	MAFN	$R^{2}: 0.71$
Putro et al. 2024^110^	10	16	23 (NinaPro DB5 Exp. 3)	22	Transformer	$R^{2}: 0.97$

The results of this study have significant implications for enhancing HCI. The high decoding performance and low decoder latency suggest that the NeuroLife EMG sleeve can provide a more natural and responsive interface for controlling prosthetic devices, assistive devices, or interacting in virtual or robotic environments^111^. In addition, the NeuroLife EMG sleeve is designed to be easier and more robust to use than other HD-EMG grid arrays, thereby improving usability without sacrificing performance^69^. While the results of this study are promising, there are several areas for future improvement. Decoder latency could potentially be reduced further by using overlapping bins^1^. While we present an initial approach at pretraining models, calibration time and data could be further reduced by using domain adaptation models with fine-tuning in session^84,112–116^. This could help promote the generalizability of the models to accommodate for individual differences in muscle physiology and movement patterns, which would further enhance the usability of the system. Furthermore, in future studies the experimental paradigm should be expanded to more dynamic tasks and interactions with external devices to better approximate real-life use scenarios.

## Conclusion

This study demonstrates the use of high-resolution EMG for hand pose identification with potential to enhance HCI. We show that the NeuroLife EMG sleeve is able to classify a wide range of movements and movement transitions reliably with minimal inference time, enabling it for use in real-time applications. Furthermore, we showed that decoder latency can be minimized by accounting for reaction time, which can help improve the responsiveness of the system. The use of pretrained models was also explored, which could potentially reduce the amount of in-session calibration data, making the system more user-friendly. Lastly, we demonstrate the feasibility of continuously estimating hand poses based on joint angle predictions using regression. The results of this study demonstrate the potential of the NeuroLife EMG sleeve and ML techniques to improve HCI by providing a more natural and responsive HMI.

### Supplementary Information

Supplementary Information 1.

Supplementary Information 2.

Supplementary Information 3.

## Data Availability

Raw data were generated at Battelle Memorial Institute. Derived data supporting the findings of this study are available upon reasonable request. Please contact the corresponding author (Nicholas Tacca) to request access (tacca@battelle.org).
