# Supplementary material for: Wearable high-density EMG sleeve for complex hand gesture classification and continuous joint angle estimation
Source: Sci Rep. 2024 Aug 9;14:18564. doi: 10.1038/s41598-024-64458-x (PMC11316006; doi:10.1038/s41598-024-64458-x)
Supplement: Supplementary file 1 — Supplementary Information 1. [file 41598_2024_64458_MOESM1_ESM.pdf]

Supplementary Information

Supplementary Table 1. Subject demographics

Subject ID	Male/Female	Age Range	Sleeve Size Session 0 (Preliminary)	Sleeve Size Session 1 (Evaluation)
1	Male	26-30	Large	Large
2	Male	26-30	N/A	Medium
3	Male	26-30	N/A	Large
4	Male	20-25	N/A	Large
5	Male	20-25	N/A	Large
6	Female	20-25	N/A	Medium
7	Female	26-30	Medium	Small
8	Male	20-25	Medium	Medium
9	Female	20-25	Medium	Small
10	Female	30-35	Large	Large

Supplementary Table 2. CyberGlove III joints. Italicized joints are calculated based on 30% of the square of the MPJ joint of the respective finger/thumb.

Channel	CyberGlove III Joint	Virtual Hand Joint
1	Thumb Rotation	Thumb Rotation
2	Thumb MPJ	Thumb MPJ
3	*Thumb IJ*	*Thumb IJ*
4	Thumb Abudction	N/A
5	Index MPJ	Index MPJ
6	Index PIJ	Index PIJ
7	*Index DIJ*	*Index DIJ*
8	Index Abduction	N/A
9	Middle MPJ	Middle MPJ
10	Middle PIJ	Middle PIJ
11	*Middle DIJ*	*Middle DIJ*
12	Middle Abduction	N/A
13	Ring MPJ	Ring MPJ
14	Ring PIJ	Ring PIJ
15	*Ring DIJ*	*Ring DIJ*
16	Ring Abduction	N/A
17	Pinky MPJ	Pinky MPJ
18	Pinky PIJ	Pinky PIJ
19	*Pinky DIJ*	*Pinky DIJ*
20	Pinky Abduction	N/A
21	Palm Arch	Palm Arch
22	Wrist Pitch	Wrist Pitch
23	Wrist Yaw	Wrist Yaw

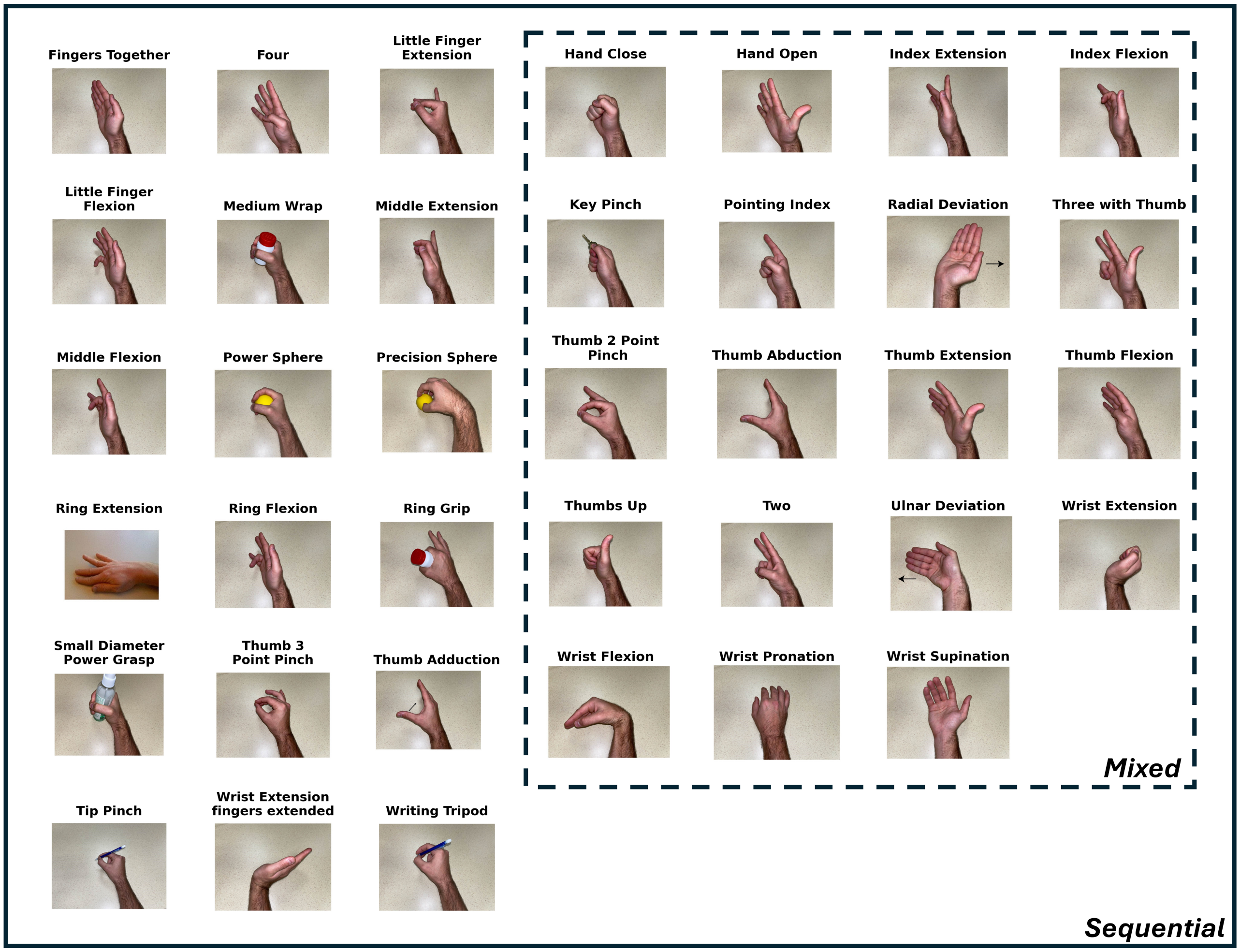

Supplementary Figure 1. Pictures of cued movements. The full 37 movements make up the sequential test set. The 19 movements in the dashed box make up the mixed test set.

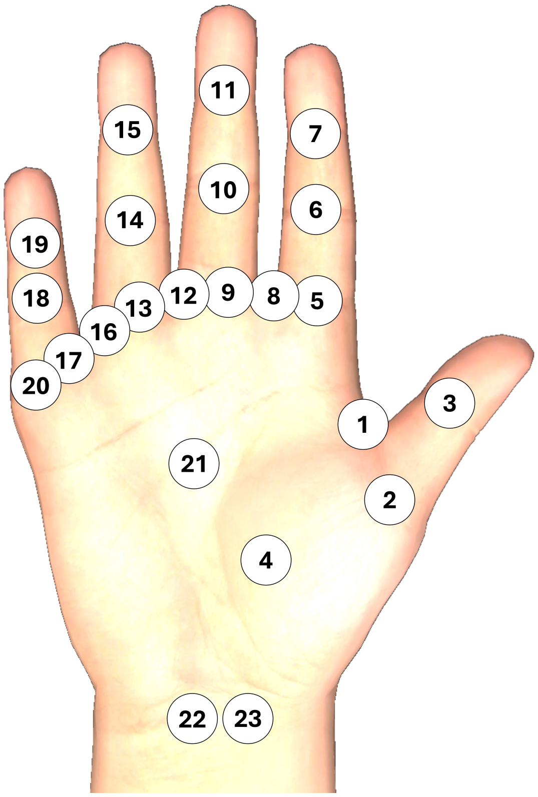

Supplementary Figure 2. Virtual hand model with CyberGlove III channels/joints represented. Refer to Supplementary Table 2 for key.

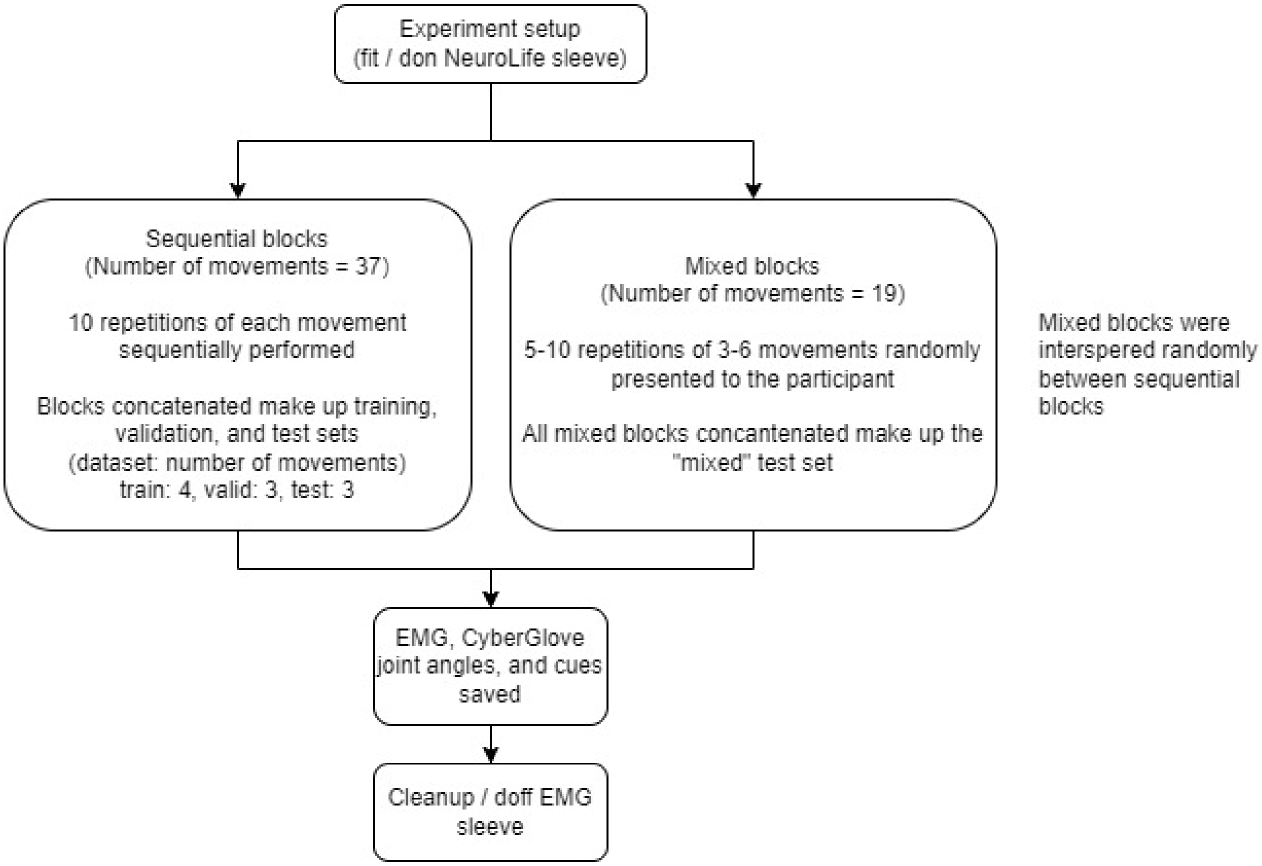

Supplementary Figure 3. **Flowchart of experiment methods and data recording blocks.**

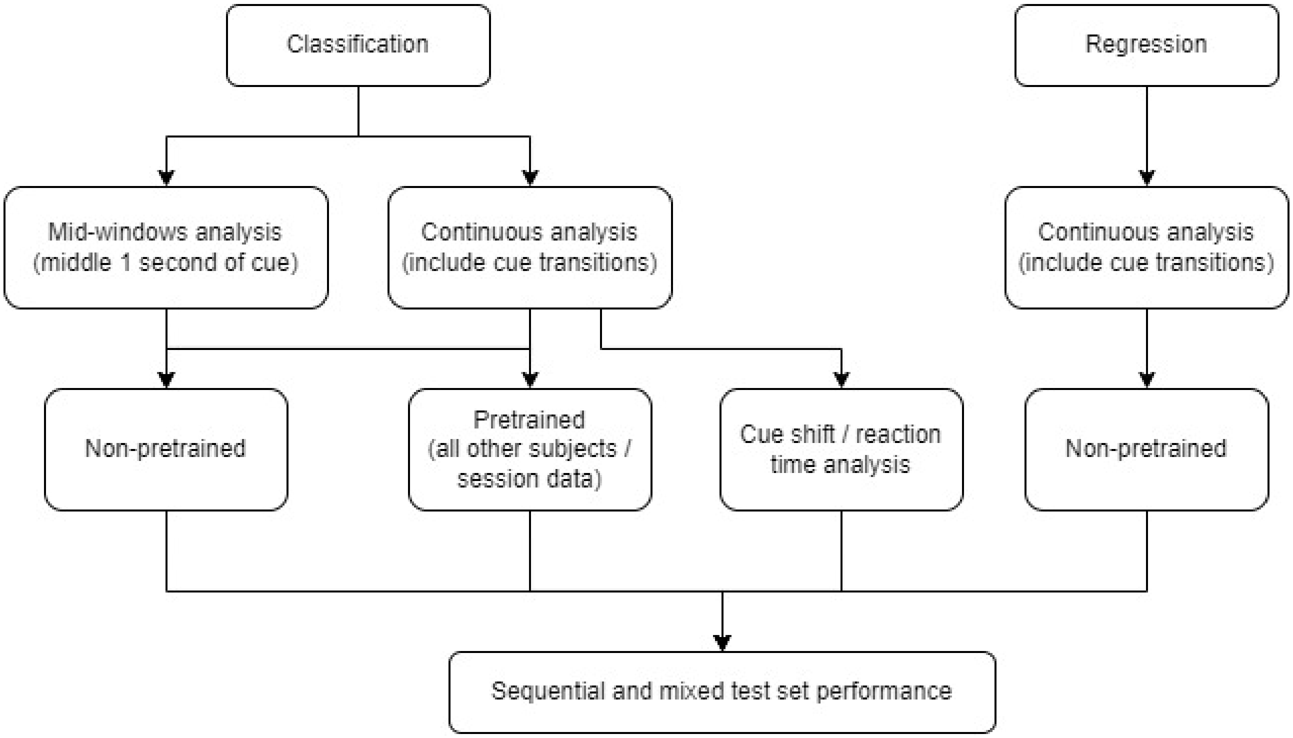

Supplementary Figure 4. **Flowchart of decoding analyses performed.**

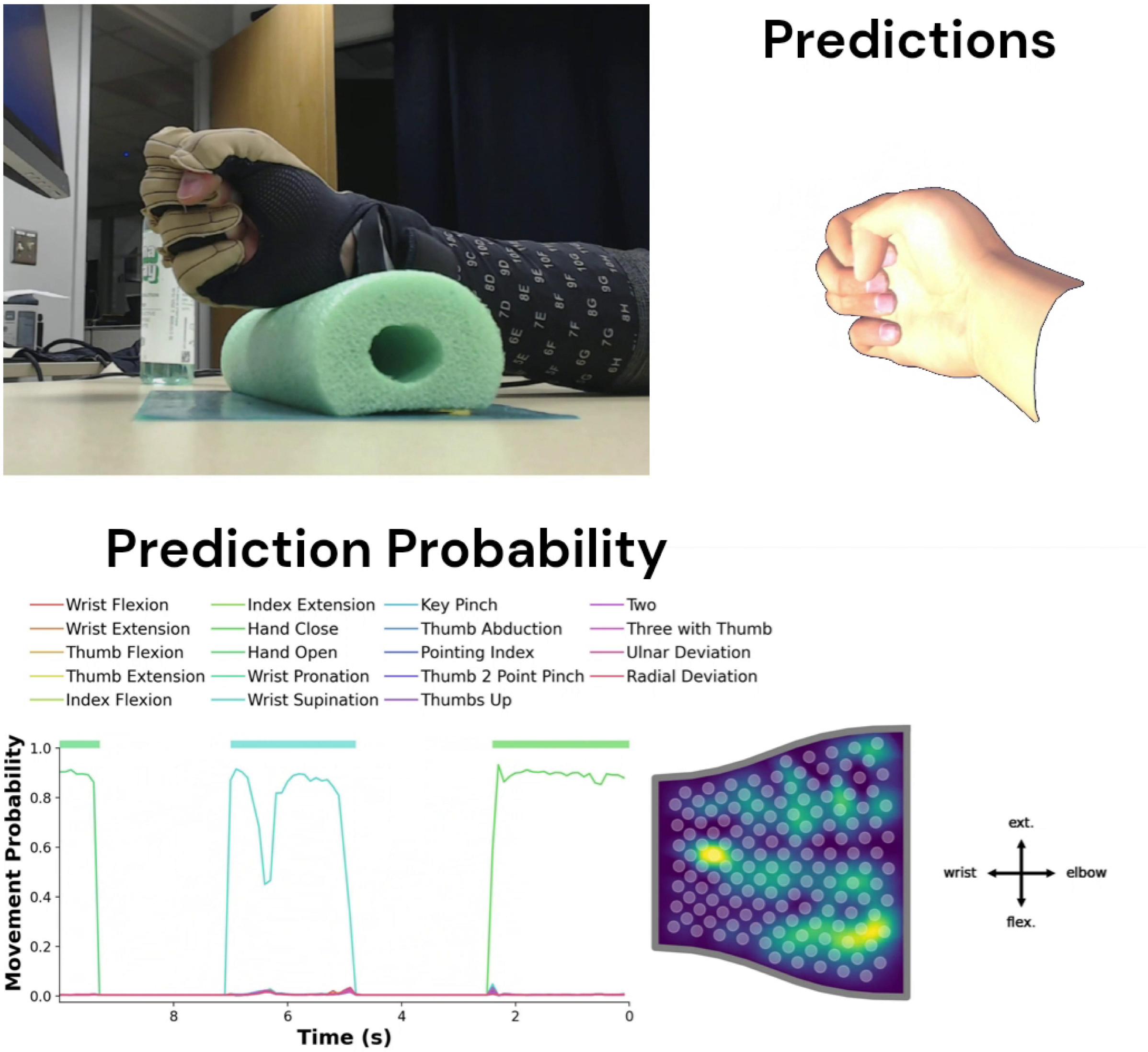

Supplementary Video 1. Simulated real-time classification video for subject 4 in the mixed dataset. A webcam video shows the true hand position during the task. The virtual hand responds to the classes predicted with a blue mask showing the ground truth labels determined by the dynamic cue shift method. A prediction probability plot shows the output of the model softmax function. Shaded rectangles above the lines indicate the ground truth labels. A sleeve heatmap shows the spatial normalized EMG RMS activity. *Note: The video speed is 2X.*

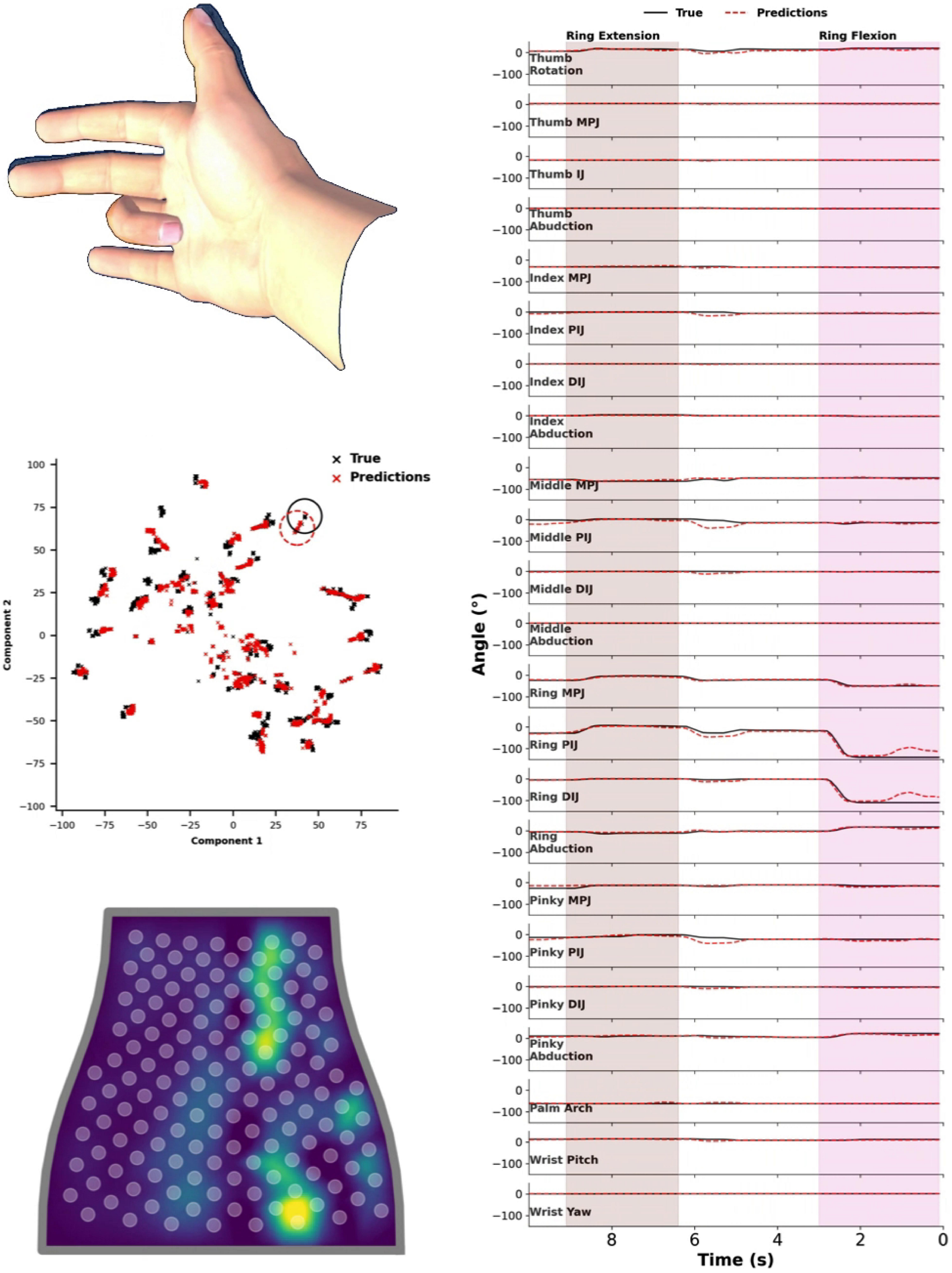

Supplementary Video 2. Simulated real-time regression video for subject 1 in the mixed dataset. A virtual hand shows the hand positions predicted by the neural network regression model. A blue mask behind the hand shows the ground truth measured with the CyberGlove III. A T-distributed stochastic neighbor embedding (TSNE) plot shows the predictions and ground truth joint angles embedded to two components. A flattened sleeve heatmap shows normalized EMG RMS activity. The full right panel shows simulated real-time joint angle predictions (red dashed) with ground truth measurements (black) across all 23 joints. *Note: The video speed is 2X.*
